# Hormone-Balancing Effect of Pre-Gelatinized Organic Maca (Lepidium peruvianum Chacon): (II) Physiological and Symptomatic Responses of Early-Postmenopausal Women to Standardized doses of Maca in Double Blind, Randomized, Placebo-Controlled, Multi-Centre Clinical Study

**Published:** 2006-12

**Authors:** H. O. Meissner, A. Mscisz, H. Reich-Bilinska, W. Kapczynski, P. Mrozikiewicz, T. Bobkiewicz-Kozlowska, B. Kedzia, A. Lowicka, I. Barchia

**Affiliations:** 1 *Faculty of Health Studies, Charles Sturt University & Therapeutic Research International, GPO Box 4792, Sydney 2001, Australia;*; 2 *Research Institute of Medicinal Plants, 27 Libelta St., 61-707 Poznan, Poland;*; 3 *Specialist Gynecology Private Clinic, Glogow, Poland;*; 4 *Specialist Gynecology Private Clinic, Poznan, Poland;*; 5 *Department of Pharmacology, Medical University, Poznan, Poland;*; 6 *Department of Primary Industry, E. Macarthur Institute, Menangle, Australia*

**Keywords:** blood biochemistry, early-postmenopause, hormones, HRT, maca (lepidium peruvianum)

## Abstract

This was a double-blind, randomized, placebo-corrected, outpatient, multi-centre (five sites) clinical study, in which a total of 168 Caucasian early-postmenopausal women volunteers (age>49 years) participated after fulfilling the criteria: follicle stimulating hormone (FSH) >30 IU/ml and estrogen (E2) <40 pg/ml levels at admission. They were randomly allocated to Placebo and Pre-Gelatinized Organic Maca (Maca-GO) treatment, according to different monthly treatment sequences scheduled for each site. Two 500 mg vegetable hard gel capsules with Maca-GO or Placebo powder were self-administered twice daily with meals (total 2 g/day) during three (Trial I; n=102) or four (Trial II; n=66) months study periods. At the baseline and follow- up monthly intervals, blood levels of FSH, E2, progesterone (PRG) and lutinizing hormone (LH), as well as serum cholesterol (CHOL), triglycerides (TRG), high- and low density lipoproteins (HDL and LDL) were measured. Menopausal symptoms were assessed according to Greene’s Score (GMS) and Kupperman’s Index (KMI). Data were analyzed using multivariate technique on blocs of monthly results in one model and Maca versus Placebo contrast in another model. A total of 124 women concluded the study. Maca-GO significantly stimulated production of E2 (*P*<0.001) with a simultaneous suppression (*P*<0.05) of blood FSH, increase (*P*<0.05) in HDL. Maca-GO significantly reduced both frequency and severity of individual menopausal symptoms (hot flushes and night sweating in particular) resulting in significant (*P*<0.001) alleviation of KMI (from 22 to 10), thus, offering an attractive non-hormonal addition to the choices available to early-postmenopausal women in the form of a natural plant alternative to Hormone Replacement Therapy (HRT) – hence, reducing dependence on hormone therapy programs.

## INTRODUCTION

Amongst a variety of traditional applications, Maca has been used for years by the native people of Peru as an energizing vegetable for maintenance of health at high altitudes in the Andes and as a dietary supplement. It has been used as a traditional remedy to treat common health problems in both men and women (1-3) as well as being used successfully to treat various, specific female-related disorders such as hormonal imbalances, menstrual irregularities, infertility, and menopausal symptoms, including hot flashes, vaginal dryness, loss of energy and libido, and depression (4, 5). There are indications (3-5), that Maca can be helpful in reducing discomfort caused by menopausal symptoms. Limited case studies on pre- (6) and postmenopausal women (7) followed by an in-depth model study on laboratory animals (8, 9), conducted in this laboratory, may support those indications. Results of laboratory studies conducted by Chacon in the late 1950’s (10) suggested that the action of Maca relies on plant sterols, which act as biochemical triggers to help the body itself to increase or reduce production of individual hormones, balancing their levels appropriate to age and gender, with this trend confirmed in studies on animal models (8, 9). Since then, numerous technical reports have been published, describing the benefits of inclusion of Maca in reducing dependence of women on hormone replacement therapy (HRT) programs (3-5).

Maca was introduced to the medical profession in the USA (4) as a non-hormonal plant preparation with potential use in anti-aging treatments, adding to a list of estrogenic herbal preparations including Soy-based products, Red Clover, Black Cohosh, Wild Yam etc. Maca preparation was classified as non-toxic for use by humans (8) at the LD50>7.5 g/kg body weight (BW), which was well above the 2 g/kg BW limit determined by the OECD (11) for dietary supplements.

Previous paper (Part I) from this series (9) reported on the biochemical and pharmacodynamic effects of pre-Gelatinized Organic Maca (Maca-GO) preparation used in a model laboratory study on ovariectomised rats. Four weeks administration of Maca-GO (250 mg/kg BW twice daily) to ovariectomised rats significantly reduced both Estrogen (E2) and Progesterone (PRG), while in non-ovariectomised, sexually-experienced female rats, the level of E2 was reduced only, with a simultaneous increase in PRG level. This trend has not confirmed earlier pilot observations on postmenopausal women (7) where levels of both E2 and PRG significantly increased with simultaneous decrease in Follicle Stimulating Hormone (FSH) level. Maca-GO showed a distinctive, anti-depressant-like and sedative effect, without disturbing cognitive functions or anxiolytic activity. This was associated with lowering of serum Cortisol (CT) and Adrenocorticotropic hormone (ACTH) levels, which was identical to the anti-depressive response observed in animal models. Maca-GO balanced the thyroid hormones (TSH and T3) to within physiologically-normal range, which was significantly lower for TSH and elevated for T3 in ovariectomised rats, but did not affect serum Iron level. This did not confirm earlier observations (8) nor the results reported in the literature (12) demonstrating the stimulating effect of Maca on absorption of Iron from the digestive tract.

On the basis of results from the pilot study on early-postmenopausal women conducted so far (7) and other in-depth biochemical and physiological observations made in laboratory models on animals (8, 9), it was reasonable to conclude that non-hormonal Maca-GO preparation exhibits hormone-balancing effects on the female organism and thus may reduce the discomfort experienced by women in the early-postmenopausal stage. This fact warranted in-depth clinical confirmation and clarification in view of some discrepancies between results obtained in the pilot study on women and observations made in laboratory models on mice and rats. Therefore, in this double blind, randomized, coordinated multi-centre, outpatient, full-scale clinical study, an attempt has been made to determine the effect of Maca-GO treatment in early-postmenopausal women, administered intermittently with placebo, during various, month-long intervals, on changes in levels of sex hormones and serum lipids. Simultaneously, symptoms of menopausal discomfort in participants were subjectively assessed using menopausal tests according to Greene and Kupperman.

## MATERIAL AND METHODS

### Maca

The plant species, its cultivation and proprietary processing have been described in detail in previous papers from this series (7, 8) and etno-botanical description with traditional and current applications was described in details by Chacon (1) and Obregon (2). Maca roots, used in production of Maca-GO preparation, represented typical distribution of three main root ecotypes: black, yellow and purple/red, observed in this cultivation area – averaging to approximately 16%, 48% and 9% respectively and 27% other colors. Dried Maca roots were processed at the National Institute of Agricultural Research (NIAR), National Agricultural University La Molina in Lima (Peru), after previous verification of their origin, organic status and scientific authenticity as cultivated organic Maca - *Lepidium peruvianum Chacon.*

Gelatinization index for starch component in the final product (99.8%) was confirmed analytically (according to the BRI Laboratory Assay, Sydney, Australia). Chemical characteristics of Maca-GO used in this study were identical to the one described previously (8, 9).

### Toxicity (LD50)

Toxicity of the batch of Maca-GO received for use in the study was determined on male and female rats according to the harmonized OECD procedure (11) reported in details in Part I of this work (9).

### Demographics

The study was conducted on groups of early-postmenopausal women selected from patients regularly visiting gynecologic clinics in two geographically-distant locations (some 150km apart), representing two different social matrices of population. Four private clinics (CP-1; CP-2, CP-3 and CP-4) were located in urban areas belonging to the municipality of Poznan (northern-west region of Poland) and one clinic (CG-1) in the municipality of Glogow (mid-west region of Poland). The first location (CP) may be broadly described as a light to medium industry-based city, with rich traditions and recorded history dating back to the ninth century, with numerous academic, research and cultural centers and for 50 years a host to various international expositions and Fairs (Poznan - 800,000 inhabitants). The second location (CG) represents a town which has been 100% rebuilt after World War II, with a mixed population (75,000), who settled in this town as a result of work-related migration based on economic needs. The population is entirely dependent on the local copper mining industry which was established in this region in early-1950s (Glogow – the largest copper mining and processing region in Europe).

### Subjects

All subjects considered for inclusion in the study were early-postmenopausal women in good overall health and fulfilling menopausal criteria set at the levels: for FSH of 30 mIU/mL level or more and E2 of 40 pg/mL levels or less. Subjects had experienced absence of menses for at least 6 months, were not on HRT, or had discontinued HRT at least 12 months before admission to the study. Subjects were excluded if they had a history of breast cancer, hyperplasia, endometrial carcinoma, or cervical neoplasia; undiagnosed abnormal vaginal bleeding; a bilateral hysterectomy; history of cardiovascular disease; liver disease; history of chronic alcoholism, medication hypersensitivity, or allergy to dietary supplement ingredients; uncontrolled addiction or severe depression; acute systemic infection or abnormal laboratory values.

As a result of screening procedures in five clinics, a total of 168 participants representing Caucasian, early-postmenopausal women volunteers aged 49 to 58 years were selected. They represented healthy subjects willing to participate in the study, of three (Trial I) and four month duration (Trial II), each addressing separate issues as per aims of the study:

**TRIAL I: A follow-up, three-month Trial, to confirm outcome of pilot study (7) and to establish residual effects of Maca-GO treatment.** This double blind, randomized, coordinated multi-centre, outpatient clinical study was established in five clinics (four in Poznan=CP and one in Glogow=CG with two different admission periods #a and #b). A total 102 women volunteers were enrolled (12 subjects in CP-1; 10 in each CP2; CP-3 and CP-4 and 24 in CG-1a and 36 in CG-1b) with five MD specialist Gynecologists being responsible for the implementation of unified experimental protocol according to coded treatment sequence, allocated at random to individual clinics, the schedule of which was known to the study coordinator only.

In one treatment, a group of women (n=62 in five clinics) was exposed to experimental protocols adopted in the pilot study (7) in which, after admission point (A), one month Placebo treatment (P) was introduced prior to the two months Maca-GO (M) treatment (A-P-M-M).

In order to observe “residual effect” of Maca treatment after Maca-GO was withdrawn from participants for one month, the second group of women (n=40 in two clinics) was given Maca-GO capsules for the two months period from the Admission point, followed by one month Placebo capsules (A-M-M-P).

All voluntarily participating women, after being assessed by a Gynecologist and confirmed on the basis of adopted analytical blood criteria as early-postmenopausal, were enrolled in this 3 month long Trial. All participants agreed to four, monthly “in-clinic” visits (at admission and then after each month of the treatment) to be physically examined by the Gynecologist, with blood sampling for hormone analysis and a simultaneous interview with the Gynecologist to assess their menopausal symptoms according to questionnaire by Greene (GMS) and Kupperman (KMI).

In the clinic CG-1, admission series CG-1a, total of 24 participating women (12 women in each treatment sequence), all four sex hormones (FSH, E2, PRG and Lutinizing Hormone – LH), as well as GMS and KMI were determined, while in admission series CG-1b (18 women in each treatment sequence), in addition to GMS and KMI, only FSH and E2 were analyzed and four blood lipid indices (Triglycerides - TRGL, Total Cholesterol - CHOL, High- and Low Density Lipoproteins - HDL and LDH) were determined.

**TRIAL II: Assessing Placebo effect when introduced in different sequence and length intervals with Maca-GO (four months multi-centre Trial).** In this double blind, randomized, coordinated multi-centre, outpatient study on 66 early-postmenopausal women, an attempt was made to determine the effect of Placebo administered intermittently with Maca-GO (Placebo as a resting period from Maca-GO treatment), on the degree of womens’ responses to Maca-GO administered in various monthly intervals during the four month treatment program. In five clinics, six individual treatment sequences were used (A-P-M-M-P; A-P-P-M-M; A-M-M-P-P; A-M-M-P-M; A-P-P-P-M; A-M-M-M-P). Eleven women were assigned to each treatment sequence.

Blood levels of FSH, E2, PGR and LH were measured and a degree of menopausal discomfort was determined according to Greene and Kupperman.

### Procedure

The protocol and amendments were approved by the Bioethics Committee of Medical Review Board in Poznan (No. 11/2004). Written informed consent was obtained from each enrolled subject regarding voluntary participation in the trial conducted under the supervision of the specialist Gynecologist, who had explained the purpose, benefits and possible risks of the study, its requirements and procedures.

In all Trials, one of the responsible investigators enrolled all patients in each clinic and randomly allocated subjects to a predetermined sequence of treatment after previous clinical confirmation of blood FSH and E2 eligibility criteria. The patients as well as research team were kept blind throughout the study.

At the admission and during further consultation visits to the Gynecologist, each eligible subject was given a complete physical and pelvic examination, blood was sampled for clinical tests and an assessment of specific symptoms describing menopausal status was made using questionnaires according to GMS and KMI. Subjects were instructed to return any unused portion of monthly volume of capsules so as to determine compliance.

### Experimental protocol

The study was carried out by specialist Gynecologists and researchers from the Research Institute of Medicinal Plants (RIMP) and Medical University in Poznan, under the strict supervision of the Study Coordinator between January 2004 and June 2005 according to experimental design previously approved by the Bioethics Committee. Encapsulation and coding of monthly doses of the appropriate treatments was conducted at the RIMP. Maca-GO or Placebo powder (sorbitol & cellulose) were encapsulated in identical-looking hard gelatin vegetable capsules (500 mg net per capsule). Capsules used in this study were kept in numbered containers for delivery to appropriate centre, according to the pre-determined experimental design known to the study coordinator only.

During a monthly interview with a Gynecologist who was one of the investigators, all participants received a numbered container with a monthly allocation of capsules to be taken as a dose 4 × 500 mg capsules (2,000 mg) daily, either Maca-GO, or Placebo. The appropriate monthly capsule doses were self-administered according to the following schedule: 2 capsules some 30 minutes before the morning meal and 2 capsules before the evening meal for the period of three (Trial I) or four months (Trial II) where four or five visits to the clinic were required respectively. The 2 g/day dose of Maca-GO was identical to the one applied in a pilot study (7) and was adopted from practical clinical experience by Muller (4, 5) who recommended such a dose for alleviation of menopausal discomfort in women involved in various therapeutic programs in clinics in the USA.

### Assays

Blood serum level of hormones was measured on monthly basis for: 17β-estradiol (E2), FSH, LH and PRG and lipid profiles (CHOL, TRGL, HDL, LDL). Indices of menopausal discomfort were determined in personal interviews by a doctor using the Menopausal Score and Index questionnaire according to Greene (7) and Kupperman (6).

Blood analyses were conducted by two clinical laboratories (for clinics in Poznan by “Labo-Med” Medical Diagnostic Centre, Pod Lipami, Poznan, Poland, and for Glogow by “Prodok” Medical Laboratory, Przylep, Poland), using officially accepted standard clinical procedures on Immulite – DPC equipment. Precision of analytical techniques in both Laboratories is monitored by National Center of Quality of Diagnostic Medical Laboratories in Poland and both Laboratories are participants in the International Quality Control RIQAS maintained by Randox Company.

### Statistical analysis

Data within each Trial were expressed as means with a linear mixed model fitted to data, allowing the comparison between groups of individuals within each month and taking into account the effects of individuals on each response variable (combined fixed and random effect). The errors were assumed to follow a Gaussian (normal) distribution. All parameters were estimated using the Restricted Maximum Likelihood (REML) estimation (13). The comparisons between groups within months were made using least significant difference (LSD) test with the differences considered significant at *P*<0.05 and highly significant at *P*<0.01 and *P*<0.001 levels.

**Greene’s and Kupperman’s scores of postmenopausal symptoms:** Total scores defined by both GMS and KMI were analyzed using the conventional analysis of variance to compare the individual groups (Menopausal status by Maca-GO treatment combinations). Weighted total scores of 21 in GMS and indexed 11 symptoms based on KMI formula were analyzed in a similar manner to the hormone data. Significant differences were tested using the least significant difference (LSD) at 5% level.

A multivariate analysis via the canonical variate technique (14) was used to relate 21 (GMS) or 11 (KMI) response variates to groups of menopausal women treated in various sequence combinations of Maca-GO & placebo treatment. Monthly data were analyzed separately. All analyses were performed using SAS statistical software (15).

## RESULTS

### Toxicity (LD50) of Maca-GO preparation

As reported in Part I of the paper (9), all animals survived the LD50 toxicity test within the range of administration 0.75 g/kg BW and 15 g/kg BW without any adverse effects noted on the basis of abnormal behavior and histopathology of internal organs (liver, spleen, pancreas and testis or ovaries).

### Subjects’ participation

The study began with total 168 subjects registered at the admission point: 102 in Trial I and 66 in Trial II.

In Trial I, total 14 subjects failed to present themselves to consulting doctors for their monthly checkup and to obtain next portion of monthly supply of capsules, with total 88 participants completing the three month study (55 in APMM group and 33 in AMMP group). Seven subjects resigned while on APMM treatment prior to conclusion of the first month on Placebo treatment, with reason given that they were discouraged after feeling no expected effect of the treatment, and seven subjects on the AMMP after the first two months on Maca treatment, admitting that they were not taking capsules while on holidays or were inconsistent with daily capsule administration routine.

In Trial II, out of 66 subjects allocated into six groups of 11 each at admission point, only 40 women satisfactorily concluded the four month Trial. In the two sequence groups: APPPM and AMMMP located in two clinics in Poznan (CP-4 and CP-5), out of the total 22 admitted participants, only two women concluded the four month Trial. Women resigned during the first and the second month participation due to admitted irregular intake of capsules; hence, both these groups were excluded from statistical analysis. Four women from APMMP and two from AMMPM sequence group failed to present themselves for monthly examination after the first or second month of study (admitting that they were not taking capsules regularly or after not feeling anticipated reduction in menopausal discomfort). Therefore, records from only 38 subjects in four treatment groups were submitted to statistical analysis in this Trial (sequence: APMMP, APPM, AMMPP and AMMPM).

### TRIAL I

Results from hormone assays (Table 1) showed that, in comparison to placebo, out of four hormones studied, a significant (*P*<0.05) decrease in FSH and a highly significant (*P*<0.001) increase in E2 were observed in sequence group APMM only, without significant changes (*P*>0.05) recorded in PRG and LH levels within the same sequence treatment. There were no significant changes recorded in any of the hormones within a sequence group AMMP (*P*>0.05), nor between Placebo and Maca-GO treatment in subjects who received Placebo after previous two months Maca-GO treatment (*P*>0.05). There were no statistically-significant differences (*P*>0.05) between the two sequence groups in levels of four hormone profiles recorded on monthly basis in any of the four blood sampling points.

**Table 1:** TRIAL I. Average Admission (A) and monthly values for hormones FSH, E2, PRG and LH levels at four sampling times (Month Model)^a^ and Placebo (P) versus Maca-GO (M) contrast (Treatment Model) for two application sequences: APMM (n=55) and AMMP (n=33) during three months long study

Hormone	Treatment	Admission (A)	End of Month 1	End of Month 2	End of Month 3	*SED*^b^	Placebo^c^	Maca	*SED*	*P*^d^

FSH (IU/ml)	APMM	60.86	61.70	56.11	54.66	4.117	61.72	55.43	3.19	<0.05
	AMMP	67.14	61.09	50.51	56.4	4.537	55.68	55.1	3.96	ns
	*SED*		6.36	6.48	6.65		6.25	5.77		
	*P*		ns	ns	ns		ns	ns		
E2 (pg/ml)	APMM	22.39	17.15	30.41	32.04	5.183	17.16	31.21	4.23	<0.001
	AMMP	17.22	22.97	30.8	24.8	5.727	24.84	27.36	4.99	ns
	*SED*		5.94	5.96	6.2		5.83	4.66		
	*P*		ns	ns	ns		ns	ns		
PRG (ng/ml)	APMM	0.425	0.494	0.392	0.496	0.201	0.510	0.472	0.156	ns
	AMMP	0.469	0.541	0.606	0.331	0.225	0.331	0.574	0.195	ns
	*SED*		0.27	0.296	0.221		0.233	0.2416		
	*P*		ns	ns	ns		ns	ns		
LH (mU/ml)	APMM	26.7	29.13	28.24	29.71	2.25	29.12	29.12	1.74	ns
	AMMP	31.55	31.67	28.29	26.18	2.477	26.18	29.98	2.18	ns
	*SED*		3.86	4.12	3.99		3.89	3.64		
	*P*		ns	ns	ns		ns	ns		

a A linear mixed model was fitted to data, allowing the comparisons between treatment groups: treatment by month differences in one model and Maca versus Placebo contrast in another model. The random effects included both individual variation and residuals. Two models may be written as follows: Month Model, Fixed (Treatment + Month + Interaction) + Random (Individuals + error); Treatment Model, Fixed (Treatment + Maca + Interaction) + Random (Individuals + error). The errors are assumed to follow a Gaussian (normal) distribution. All parameters were estimated using the Restricted Maximum Likelihood (REML) estimation. The differences between treatments within each month and between Maca and placebo were tested using least significant difference (LSD) test at 5% and 1% significance levels;

b *SED*, Standard error of differences;

c Capital letters attached to the means indicate significant difference between values in columns. Small letters indicate significant differences between values in rows.

d *P*, Probabilities of significance. ns, not significant at *P*>0.05; *P*<0.05, significance at 5% probability level; *P*<0.01, significance at 1% probability level; *P*<0.001, significance at 0.1% probability level.

In comparison to the admission point, after two months of Maca-GO treatment, there was statistically-significant (*P*<0.05) reduction in blood TRGL and LDL concentrations, at a simultaneous significant (*P*<0.05) increased in HDL levels (Table 2). Comparing to Placebo, one month Maca-GO intake was not long enough for the treatment to produce statistically-significant changes in lipid indices, but after two month a significant (*P*<0.05) increase in HDL concentration was observed.

**Table 2:** TRIAL I. Average Admission (A) and monthly values for Lipids: Cholesterol (CHOL), Triglycerides (TRGL), High Density Lipoproteins (HDL) and Low Density Lipoproteins (LDL) levels at four sampling times (Month Model)1 and Placebo (P) versus Maca-GO (M) contrast (Treatment Model) for two application sequences: APMM (n=18) and AMMP (n=18) during three months long study^a^

Measurement	Treatment	Admission (A)	End of Month 1	End of Month 2	End of Month 3	*SED*^b^	Placebo	Maca	*SED*	*P*^c^

CHOL(mg/100ml)	APMM	255.2	234.5	224.1	218.6	10.53	234.5	222.3	8.1	ns
	AMMP	236.9	236.0	226.9	238.6	7.87	238.6	231.6	6.9	ns
	*SED*		15.13	15.17	16.3		14.43	14.53		
	*P*		ns	ns	ns		ns	ns		
TRGL (mg/100ml)	APMM	130.0	125.2A	107.2AB	83.8B	19.7*	125.2	98.8	15.5	ns
	AMMP	137.4	130.5	123.7	129.7	14.5	129.7	127.0	12.3	ns
	*SED*		26.4	26.63	28.4		24.7	25.3		
	*P*		ns	ns	ns		ns	<0.05		
HDL (mg/100ml)	APMM	67.07	59.54B	62.63AB	73.02A	5.39*	59.54	65.68	3.91	ns
	AMMP	61.54	66.38AB	69.83A	62.65B	3.13*	62.65B	68.07A	2.7	<0.05
	*SED*		5.42	5.73	6.62		5.07	5.42		
	*P*		ns	ns	ns					
LDL (mg/100ml)	APMM	187.7	171.7	165.0	149.5	13.8	171.7	157.2	9.8	ns
	AMMP	169.2	166.8	155.2	170.6	8.0	170.6	160.7	7.1	ns
	*SED*		15.1	16.1	18.6		14.4	15.4		
	*P*		ns	ns	ns		ns	ns		

a For explanation see Table 1;

b Standard error of differences;

Asterisk (*) indicates existence of significant differences between monthly measurements within the same treatment sequence group. Values marked with unlike capital letters are considered statistically significant at *P*<0.05;

c Significance probabilities: ns = not significant at *P*>0.05; *P*<0.05 = significance at 5% probability level; *P*<0.01 = significance at 1% probability level; *P*<0.001 = significance at 0.1% probability level.

### TRIAL II

Placebo introduced at the start of the Trial has slightly (*P*>0.05) lowered the FSH in group APMMP in relation to Admission point, however taking Placebo for one month again after two months Maca-GO treatment, resulted in a distinctive and highly significant (*P*<0.01) increase in concentration of this hormone in relation to both Admission point and both monthly measurements on Maca-GO (Table 3). Two month Placebo at the beginning of the Trial or during the last two months following two months of Maca-GO treatment had no significant (*P*>0.05) effect on FSH results – although Maca-GO had a distinctive tendency to lower level of FSH. Women in sequence Group AMMPM showed highest degree of reduction in FSH after two months of Maca-GO intake (from 60 to 28.8 IU/ml) with one month Placebo treatment significantly (*P*<0.05) increasing blood FSH and after returning to Maca-GO treatment for another month giving a distinctive, although not statistically significant (*P*>0.05) reduction in this hormone. In all sequence groups, two months of Maca-GO treatment was more pronounced as compared to one month intake of capsules only.

**Table 3:** TRIAL II. Average Admission (A) and monthly values for FSH, E2, PRG and E2 levels at five sampling points (Month Model)^a^ and Placebo (P) versus Maca-GO (M) contrast (Treatment Model) for five application sequences (n=42) during the four months long study: APMMP (n=7), APPMM (n=11), AMMPP (n=11) and AMMPM (9)

Hormone	Treatment^b^	Admission (A)	End of Month 1	End of Month 2	End of Month 3	End of month 4	*SED*^c^	Placebo	Maca	*SED*	*P*^d^

FSH (IU/ml)	APMMP	A 85.73	P^e^ 78.48	M 70.39	M 64.94	P 97.88	10.10	88.18	68.12	6.95	<0.01
	APPMM	A 64.62	P 70.54	P 71.28	M 65.60	M 60.66	8.08	70.91	63.56	5.6	ns
	AMMPP	A 69.03	M 62.01	M 57.27	P 53.63	P 59.22	7.46	56.42	59.64	5.29	ns
	AMMPM	A 60.06	M 49.60	M 28.77	P 53.67	M 40.35	11.46	53.67	40.47	6.81	ns
	*SED*		17.301	17.348	17.371	19.208		16.565	15.618		
	*P*		ns	<0.05	ns	<0.05		<0.05	ns		
E2 (pg/ml)	APMMP	30.42	35.73	37.26	31.20	35.93	28.34	35.87	34.23	18.84	ns
	APPMM	19.11	8.33	15.14	45.72	58.43	22.19	11.73	50.95	15.36	<0.05
	AMMPP	8.62	12.08	25.3	25.61	17.14	21.60	21.13	18.69	15.05	ns
	AMMPM	30.19	39.94	77.25	54.27	87.97	21.5	54.27	65.69	16.47	ns
	*SED*		31.661	28.375	29.016	35.96		18.992	19.124		
	*P*		ns	<0.05	ns	ns		<0.05	<0.05		
PRG (ng/ml)	APMMP	0.426	0.659	0.574	0.484	0.351	0.259	0.505	0.529	0.181	ns
	APPMM	0.201	0.233	0.295	0.209	0.286	0.250	0.264	0.241	0.174	ns
	AMMPP	0.225	0.233	0.373	0.358	0.302	0.233	0.330	0.303	0.163	ns
	AMMPM	0.561	0.432	0.4309	0.557	0.370	0.242	0.557	0.431	0.187	ns
	*SED*		0.241	0.237	0.240	0.270		0.188	0.165		
	*P*		ns	ns	ns	ns		ns	ns		
LH (mU/ml)	APMMP	21.62	30.69	26.81	28.78	28.01	4.18	29.35	27.74	2.9	ns
	APPMM	43.79	38.92	38.91	34.05	32.41	3.985	38.91	33.38	2.74	<0.05
	AMMPP	36.66	32.69	32.58	30.03	32.5	3.678	31.27	32.64	2.59	ns
	AMMPM	26.43	30.65	24	22.35	28.3	3.77	22.35	27.58	2.91	ns
	*SED*		8.6	8.566	8.622	8.805		6.203	6.035		
	*P*		ns	ns	ns	ns		<0.05	ns		

a For explanation see Table 1;

b In this Table (Trial II), participants in the two sequence groups: APPPM and AMMMP have been excluded from analysis of data due to the two women only, out of the total 22 admitted women concluded the four months Trial located in two separate clinics in Poznan (CP-4 and CP-5);

c *SED*, Standard error of differences;

d *P*, Probabilities of significance. ns, not significant at *P>0.05*; *P<0.05*, significance at 5% probability level; *P<0.01*, significance at 1% probability level; *P<0.001*, significance at 0.1% probability level;

e Letters preceding the values in columns for consecutive blood sampling points within the individual hormone, indicate hormone level as recorded after one month of treatment with either Placebo (P) or Maca-GO (M) within the relevant four treatment sequences analyzed in this Trial. Letters’ pattern for the E2, PRG and LH are identical to those as marked in the FSH data block.

After exposure to Maca-GO intermittently with the Placebo, levels of E2 showed opposite trends to the one observed in FSH blood contents, which resulted in Maca-GO significantly (*P*<0.05) increasing E2 values in sequence group APPMM and nearly-significantly in group AMMPM (*P*>0.05). Two months Maca-GO treatment magnified this trend in comparison to one month of treatment.

There were no significant (*P*>0.05) differences recorded in PRG concentrations when Maca-GO was intermittently introduced to women in various sequence patterns and for different length of time. Maca-GO treatment significantly (*P*<0.05) lowered LH in a sequence group where two months Placebo treatment was followed by Maca-GO application.

There were significant differences (*P*<0.05) detected between sequence groups within both Placebo (FSH, E2, and LH) and Maca-GO (E2 only) blocks of data, with sequence groups APMMP and AMMPM representing clinics located in Poznan and the other two in Glogow which shows differences existing in dynamics of responses of participating women to Maca-GO in two geographically-distant locations.

### Kupperman’s Menopausal Index (KMI) and Greene’s Menopausal Score (GMS)

Responses of participants to two questionnaires collected by Gynecologists were computed to derive menopausal index according to Kupperman (KMI) and menopausal score according to Greene (GMS), allowing for an evaluation of menopausal discomfort expressed as sums of recorded points derived from scores of individually-assessed symptoms.

In Trial I, as compared to the Admission point, after one month Placebo treatment, before introduction of Maca-GO (APMM), there was already statistically-high significant reductions (*P*<0.001) in total KMI and GMS values(Table 4). Similar (*P*<0.001) reductions in feelings of discomfort, typically observed in early- postmenopausal stage, were recorded in women receiving Maca-GO treatment immediately after the admission point (AMMP). After the second month of Maca-GO treatment, in both sequence groups, there was further significant (*P*<0.001) lowering in both KMI and GMS total values. On the other hand when Placebo was introduced after two months of Maca-GO treatment, there was an overall statistically-high increase (*P*<0.001) in total KMI and GMS values, these being a reflection of a significant increase (between *P*<0.009 and *P*<0.001 - depending on the symptom) in the severity of individual menopausal symptoms as demonstrated in Tables 5 and 6.

**Table 4:** TRIAL I. Dynamics of monthly changes in total values from symptoms determined according to Kupperman’s Menopausal Index (KMI) and Greene’s Menopausal Score (GMS) recorded between Admission point (A), at three monthly sampling points (Month Model)^a^ and Placebo (P) versus Maca-GO (M) contrast (Treatment Model) according to two treatment sequences applied in Trial I (for APMM n=55 and for AMMP n=33)

Treatment	Admission	Month 1	Month 2	Month 3	*SED*	Placebo	Maca	*SED*^b^	*P*^c^

Kupperman’s Menopausal Index (KMI)									
APMM	26.61	22.04	12.83	7.52	1.13	22.04	10.17	1.16	<0.001
AMMP	27.53	14.09	8.50	19.14	1.25	19.14	11.3	1.28	<0.001
*SED*		1.68	1.68	1.68		1.7	1.7		
*P*		<0.01	<0.01	<0.001		ns	ns		
Greene’s Menopausal Score (GMS)									
APMM	21.42	17.74	12.26	8.33	0.92	17.74	10.29	0.89	<0.001
AMMP	25.92	14.18	9.71	14.82	0.98	14.82	11.95	0.94	<0.01
*SED*		1.68	1.68	1.68		1.73	1.56		
*P*		<0.05	ns	<0.001		ns	ns		

a For explanation see Table 1;

b *SED*, Standard error of differences;

c *P,* Probabilities of significance. ns, not significant at *P>0.05*; *P<0.05*, significance at 5% probability level; *P<0.01*, significance at 1% probability level; *P<0.001*, significance at 0.1% probability level.

**Table 5:** TRIAL I. Monthly changes in values of individual symptoms determined according to Kupperman’s Menopausal Index (KMI) recorded between Admission (A), and at three monthly sampling points (Month Model)^a^ with one month Placebo (P) followed by two months Maca-GO (M) contrast according to the sequence APMM (at n = 55) applied in Trial I

Code#	Symptom in KMI	Admission A	After 1m P	After 1m M	After 2m M	± *SED*	*P*	Placebo	Maca-GO	± *SED*^b^	*P*^c^

K-1	Hot flushes	3.94	3.17	2.16	1.64	0.580	0.002	1.761	0.651	0.152	<0.001
K-2	Excessive sweating	2.41	1.24	1.11	0.85	0.276	<0.001	1.391	0.673	0.162	<0.001
K-3	Interrupted sleep	1.98	1.15	0.75	0.78	0.267	<0.001	1.196	0.605	0.144	<0.001
K-4	Nervousness	2.63	1.63	1.18	0.61	0.237	<0.001	1.630	0.802	0.145	<0.001
K-5	Depression	1.24	0.61	0.45	0.46	0.197	<0.001	0.826	0.619	0.160	0.198
K-6	Losing body balance	0.87	0.39	0.32	0.32	0.186	0.006	0.696	0.395	0.125	0.017
K-7	General weakness	1.24	0.72	0.68	0.56	0.201	0.011	1.239	0.874	0.161	0.025
K-8	Joint pain	1.02	0.65	0.70	0.69	0.208	0.001	1.000	0.719	0.158	0.079
K-9	Headaches	1.20	0.61	0.45	0.34	0.188	0.009	0.717	0.452	0.132	0.047
K-10	Heart palpitations	1.02	0.46	0.32	0.22	0.185	<0.001	0.543	0.336	0.131	0.116
K-11	Numbness hands & legs	0.57	0.39	0.20	0.20	0.190	<0.001	0.543	0.336	0.135	0.126
Total value for KMI symptoms		26.61	22.04	12.83	7.52	1.13	<0.001	22.04	10.170	1.408	<0.001

a For explanation see Table 1;

b *SED*, Standard error of differences;

c P, Probabilities of significance. ns, not significant at *P>0.05*; *P<0.05*, significance at 5% probability level; *P<0.01*, significance at 1% probability level; *P<0.001*, significance at 0.1% probability level. Scoring index: 0, symptom not experienced; 1, occasionally; 2, often; 3, very often. Kupperman’s Indexing Factors: K-1, ×4; K-2 to K-5, ×2; the remaining, ×1.

**Table 6:** TRIAL I. Monthly changes in values of individual symptoms determined according to Greene’s Menopausal Score (GMS) for APMM sequence treatment (n=55) in early-postmenopausal women recorded between Admission (A), and three monthly sampling points (Month Model)^a^ with one month Placebo (P) followed by two months Maca-GO (M) contrast (Treatment Model)1 according to the sequence applied in Trial I

Individual Menopausal Symptoms’ Scores according to Greene										
Symptom	Admission A	After Month 1 (P)	After Month 2 (M)	After Month 3 (M)	±*SED*^b^	*P*^c^	Placebo	Maca	±*SED*^b^	*P*^c^

Abnormally-fast heart rate	0.66	0.45	0.35	0.28	0.149	0.094	0.455	0.315	0.118	0.240
Nervousness	1.51	1.68	1.00	0.76	0.200	<0.001	1.682	0.879	0.169	<0.001
Difficulty falling asleep	1.15	1.12	0.77	0.52	0.212	0.009	1.121	0.646	0.169	0.006
Excessive alertness	1.33	1.33	1.00	0.55	0.175	<0.001	1.333	0.776	0.147	<0.001
Sudden feeling of anxiety	0.82	0.70	0.45	0.45	0.193	0.145	0.697	0.450	0.169	0.147
Difficulty concentrating	0.97	1.09	0.94	0.41	0.182	0.002	1.091	0.675	0.153	0.008
Feeling of tiredness/lack of energy	1.39	1.54	1.32	0.72	0.205	<0.001	1.545	1.023	0.173	0.003
Lack of interest	0.36	0.33	0.26	0.21	0.129	0.612	0.333	0.233	0.111	0.037
Unhappy/depressed	1.00	0.94	0.74	0.45	0.201	0.030	0.939	0.595	0.174	0.050
Excessive crying	0.45	0.27	0.23	0.24	0.129	0.263	0.273	0.234	0.109	0.721
Irritability	1.27	1.15	0.84	0.65	0.189	0.005	1.152	0.747	0.165	0.016
Loss of consciousness	0.61	0.88	0.45	0.41	0.170	0.030	0.879	0.433	0.147	0.003
Nervous tension	0.72	0.59	0.52	0.41	0.156	0.238	0.591	0.465	0.133	0.345
Numbness/“pins & needles”	0.67	0.61	0.45	0.31	0.183	0.207	0.606	0.381	0.153	0.144
Headaches	0.97	0.85	0.58	0.34	0.206	0.014	0.848	0.463	0.167	0.023
Muscle and joint aches and pains	1.24	1.18	0.97	0.62	0.224	0.027	1.182	0.794	0.193	0.047
Loss of feeling in feet & hands	0.45	0.36	0.19	0.24	0.148	0.284	0.364	0.217	0.123	0.239
Difficulty breathing	0.27	0.12	0.16	0.10	0.097	0.303	0.121	0.132	0.073	0.879
Hot flushes	1.73	1.73	1.10	0.52	0.257	<0.001	1.727	0.807	0.210	<0.001
Excessive night sweating	1.45	1.39	1.06	0.48	0.253	<0.001	1.394	0.774	0.203	0.003
Loss of interest in sex life	1.06	1.12	0.84	0.60	0.200	0.036	1.121	0.712	0.173	0.020
TOTAL GMS	20.09	19.45	13.36	8.15	1.869	<0.001	19.45	10.76	0.16	<0.001

a For explanation see Table 1;

b *SED*, Standard error of differences;

c *P*, Probabilities of significance. ns, not significant at *P>0.05*; *P<0.05*, significance at 5% probability level; *P<0.01*, significance at 1% probability level; *P<0.001*, significance at 0.1% probability level.

Taking APMM sequence group as an example, after Placebo administration for a one month period immediately after the Admission point (prior to Maca-GO treatment), a significant (*P*<0.001) reduction in severity of most of the 11 KMI symptoms was observed, except for the two: hot flushes and numbness in hands & legs (Table 5). With introduction of Maca-GO to participants after previous Placebo treatment, out of 11 symptoms, there was a distinctive, almost significant (*P*>0.05) lowering effect recorded in hot flushes values only, with other symptoms only slightly, not significantly (*P*>0.05) reduced. However two months of Maca-GO treatment in APMM sequence group provided the most pronounced, highly significant (*P*<0.001) reduction of hot flushes and nervousness as compared to both Admission point and Placebo treatment, while index values in other symptoms, although noticeably lowered, were not statistically confirmed (*P*>0.05).

Analyzing results for the same sequence group according to the questionnaire by Greene, which comprises 21 questions (Table 6), as compared to Placebo after one month of Maca-GO treatment, the most distinctive and highly significant (*P*<0.001) reduction in absolute GMS values was observed in hot flushes, nervousness, difficulties in falling asleep, irritability and excessive alertness. Extending Maca-GO application for another month resulted in further highly significant (*P*<0.001) lowering in the GMS values for the following symptoms: lack of energy, tiredness, excessive night sweating, hot flushes and excessive alertness. It appears that of all 21 GMS symptoms, the most sensitive in both the degree of reduction in absolute GMS value for individual symptom and the level of statistical significance (*P*<0.001), were the following: hot flushes, excessive sweating, nervousness, excessive alertness, lack of energy/feeling of tiredness and to a lesser degree (*P*<0.01) irritability and headaches.

In Trial II, as already observed in Trial I, the second month of Maca-GO treatment, magnified positive effect on alleviation of menopausal symptoms as measured by either KMI and/or GMS. Differences between Placebo and Maca-GO treatments were more pronounced in the KMI as compared to GMS (Table 7). The GMS values showed significant (*P*<0.01) effect of Placebo in one sequence group only (APPMM) while recorded KMI values showed highly significant (*P*<0.001) differences between Placebo and Maca-GO treatment in all sequence groups.

**Table 7:** TRIAL II. Dynamics of overall monthly changes in total values from individual symptoms determined according to Kupperman’s Menopausal Index (KMI) and Greene’s Menopausal Score (GMS) recorded between Admission point (A), at four monthly sampling points (Month Model)^a^ and Placebo (P) versus Maca-GO (M) contrast (Treatment Model) according to four intermittent sequence of Placebo application during four month study as per Trial II & III (n=42)

Treatment	Admission A	After Month 1 P	After Month 2 P	After Month 3 M	After Month 4 M	*SED*^b^	Placebo	Maca	*SED*	*P*^c^

Kupperman’s Menopausal Index (KMI)										
OPMMP	21.56	14.44	8.44	7.67	16.33	1.62	15.39	8.06	1.38	<0.001
OPPMM	30.40	15.20	24.00	10.80	6.99	1.69	19.60	9.65	1.31	<0.001
OMMPP	29.64	13.09	9.45	14.88	19.58	1.52	17.21	11.27	1.30	<0.001
OMMPM	25.27	14.59	9.64	17.77	11.32	1.47	17.77	11.85	1.25	<0.001
*SED*		3.52	3.52	3.53	3.59		3.47	3.47		
Greene’s Menopausal Score (GMS)										
OPMMP	25.00	19.11	12.67	10.00	8.00	1.64	13.56	11.33	1.52	ns
OPPMM	25.80	10.90	8.60	8.90	3.30	1.55	9.75	6.10	1.44	<0.01
OMMPP	30.91	12.45	10.91	13.00	7.18	1.48	10.09	11.68	1.37	ns
OMMPM	26.91	18.64	15.91	16.36	12.73	1.48	16.36	15.76	1.58	ns
*SED*		4.28	4.28	4.28	4.28		4.35	4.04		

a For explanation see Table 1;

b *SED*, Standard error of differences;

c *P*, Probabilities of significance. ns, not significant at *P>0.05*; *P<0.05*, significance at 5% probability level; *P<0.01*, significance at 1% probability level; *P<0.001*, significance at 0.1% probability.

## DISCUSSION

### The effect of Maca-GO on hormonal balance in early-postmenopausal women

According to clinical definition established by WHO, post-menopause is characterized by increased levels of gonadotropins and reduction in estrogens, with low level or absence of LH (16). Lowering of estrogen is assumed to trigger depression observed in women entering menopause, therefore, hormone therapy - through influencing an increase in blood and brain serotonin levels, particularly when combined with antidepressant medication - may alleviate stages of depression and symptoms of mood swing which are commonly observed in menopausal women (17). However, in view of the side-effects observed in women using HRT which include high blood pressure, heart complications, obesity etc. it is apparent that women are opting for non-hormonal programs, or using phyto-hormone sources such as phytoestrogens to help reduce discomfort of menopause and achieve daily comfort at this stage of life (18).

The E2 levels of 30 pg/ml and above are considered as adequate for woman (with average 60-75 pg/ml) to avoid symptoms of discomfort characteristic to menopause (19). In this study levels of E2 were below 30 pg/ml – typically indicating postmenopausal stage with impaired ovarian function (16). It is reasonable to assume that Maca-GO, although it contains no plant hormones (10), stimulated and/or contributed to the regulatory mechanism responsible for optimizing ovarian function and secretion of the quantity of estrogen – in some cases– well above the 30pg/ml considered as desired minimum (19). This may contribute to reduction in menopausal discomfort in women taking Maca-GO, avoiding problems linked to mental, physiological and physical symptoms indicated by results as measured in GMS and KMI and expressed by overall values of obtained scores (Tables 4, and 7).

Overall positive responses of participants in this study may be supported by previous pilot observations on early-postmenopausal women (7) and in-depth biochemical and physiological observations made in bioassays using sexually-experienced (8) and ovariectomised laboratory animals (9), confirming an existence of positive relationship between Maca-GO treatment and its hormone balancing function in early-postmenopausal women.

### Placebo effect in Maca-GO treatment

When Placebo was introduced for one month prior to daily dose of 2 g Maca-GO self-administered by women for two consecutive months (group APMM -55 women), there was a high degree of responses recorded by individual participants in the following key menopausal indicators, expressed either in relation to the Admission point (%A) or as Placebo-corrected values (%P) for: FSH (89%A and 78%P), E2 (80%A and 72%P), GMS (100%A and 76%P) and KMI (96%A and 85%P). As judged by absolute results (Tables 1, 3, 4 and 7) and lower %P values in relation to %A, there was a distinctive Placebo effect observed in this study, as shown by responses of early-postmenopausal women to one month Placebo treatment, which was similar, but reduced in magnitude, to the effect observed after administration of Maca-GO. Extending Placebo treatment for another month reduced or eliminated the earlier (one month) Placebo effect on women, while the effect of Maca-GO was magnified in both absolute and relative terms.

**Figure 1:**
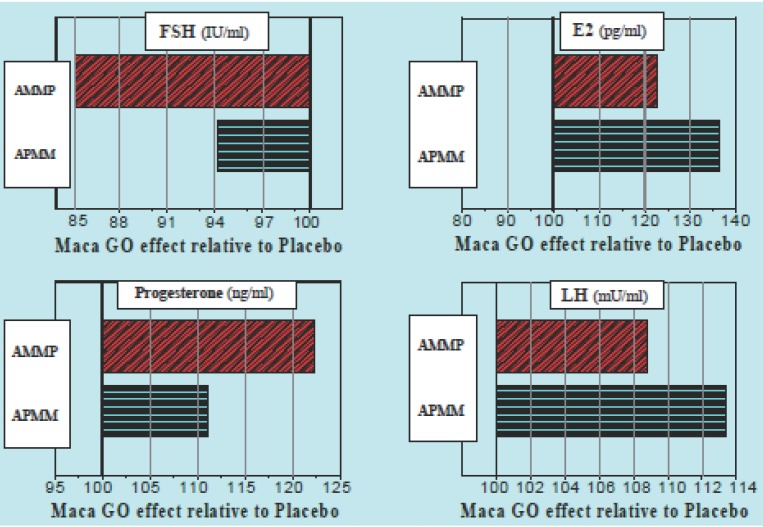
TRIAL I: Placebo-corrected relative values (*P*= 100%) for four hormones (FSH, E2, PRG and LH) in two treatment sequences (APMM and AMMP) showing different effect of Placebo, depending on the time in the sequence during the three months Trial, when Placebo was introduced prior to, or after two months Maca-GO administration to early-postmenopausal women volunteers (n=55 & n=33 respectively). *P*<0.05 = statistical significance at *P*<0.05 level.

When results from the second sequence group (AMMP – 33 women) were expressed in a similar manner to the first treatment group, generally lower responses in calculated values were obtained: for FSH (93%A and 63%P), E2 (87%A and 66%P), GMS (90%A and 84%P) and KMI (96%A and 78%P). The lower degree of responses of women to the Placebo administered after Maca-GO treatment, compared to Placebo introduced prior to the two months of Maca-GO use, may confirm an existence of a residual effect of Maca-GO treatment during the first month after its withdrawal when Placebo was used instead - the trend clearly demonstrated in Figure 1 on an example of four hormones: FSH, E2, PRG and LH.

**Figure 2:**
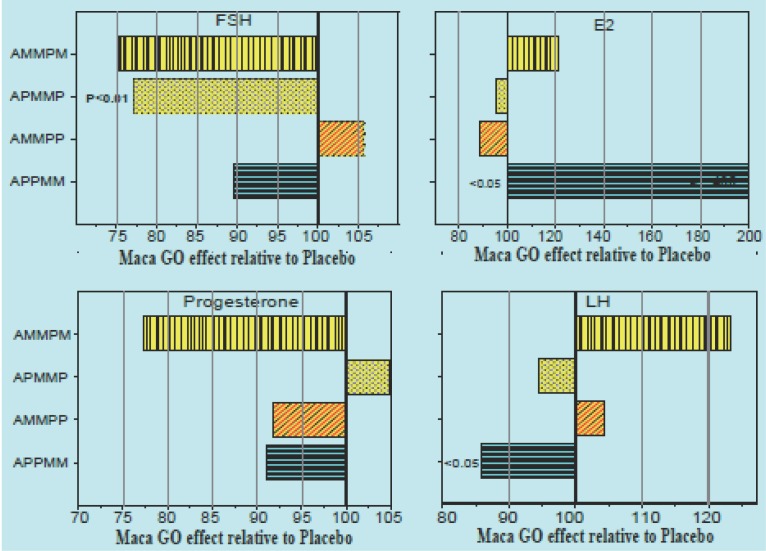
TRIAL II: Placebo-corrected values (*P*=100%) for four hormones: FSH (IU/ml), E2 (pg/ml), PRG (ng/ml) and LH (mU/ml) in four treatment sequences (AMMPM, APMMP, AMMPP and APPMM) showing different effect of Placebo on the outcome of Maca-GO treatment (M), depending on the time in the sequence during the four months Trial when Placebo (P) was administered to early-postmenopausal women volunteers (n= 40).

**Figure 3:**
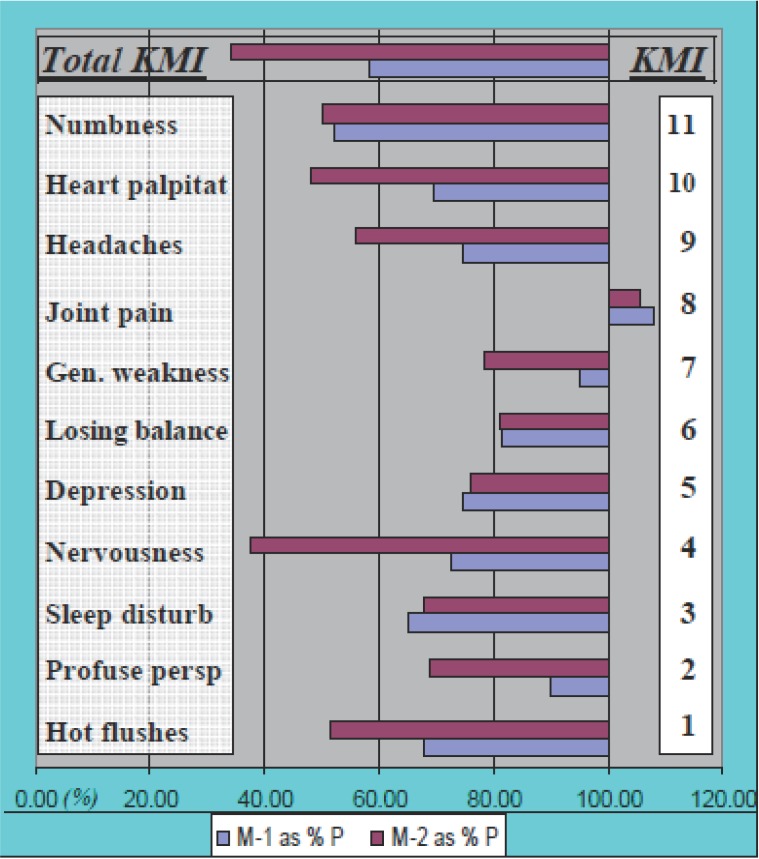
TRIAL I: Comparison of Placebo Corrected (%P) results in relative values for individual symptoms comprising Total Kupperman’s Menopausal Index (KMI) in response of early- postmenopausal women (n=55) to two consecutive monthly periods of Maca-GO application (M-1 = for one month and M-2 = for 2 months) administered after one month Placebo (P) treatment in a sequence treatment APMM (results for M-1 and M-2 are expressed as a percentage of values recorded after one month P administration).

When in Trial II results were expressed as a percentage of Placebo introduced in different sequence patterns concurrently with Maca-GO treatment (Figure 2), it was apparent that: there was different magnitude of relative responses of women to Maca-GO depending on whether Placebo was introduced prior to or after previous Maca-GO treatment; irrespective of the sequence in which Placebo was introduced during the four month trial, two months of Maca-GO application magnified the therapeutic effects of the treatment. Similar trends were observed in total GMS and KMI values (Table 4), as well as on the example of individual menopausal symptoms summarized in Figure 3.

It seems appropriate to express results attributed to full Maca-GO effect on women after two months treatment and calculate Placebo corrected values against results recorded after two month of Placebo treatment, when Placebo effect based on emotional factors is expected to be greatly reduced or eliminated in most of the measurements.

### Toxicity and LD50 for Maca-GO

Toxicity results obtained in laboratory model using rats (8) showed the value LD50>7.5 g of Maca-GO per kg body weight (BW). However, depending on the test laboratory animal being used, applied doses were between 0.75 g/kg BW through to 7.5 g/kg BW (rat’s model) to 15g/kg BW (mice model). Within the above range up to 15 g Maca-GO per kg BW (the highest dose possible to be applied in the study), no detectable negative physiological, clinical, histo-pathological, nor toxic effects existed which could be attributed to the used doses of Maca-GO and administered to rats and/or mice during model laboratory bioassays (9). Therefore, the LD50>15 g/kg BW was considered as the value for Maca-GO preparation. Such a value is considerably higher than the 2 g/kg BW limit adopted by the OECD (11) as non toxic and safe for use as dietary supplement, and therefore, Maca-GO fully conforms to such a classification as a “non toxic” with a “safe for human consumption” status.

### Distinction between Maca-GO and other commonly used herbal therapies

Several herbal alternatives have already gained acceptance by medical, health practitioners and phyto-pharmaceutical industries for potential substitution for synthetic hormones used in hormone therapies. Positive effect of soy and red clover extracts is attributed to action of flavonoids with biochemical structure resembling steroids in human hormones (20) and may induce some side effects similar to those observed after synthetic hormones are used. It has been reported that Maca preparations tend to be more effective for menopausal patients as compared to treatment with phyto-estrogenic herbs (4). Maca contains no hormones or their precursors and its action is linked to a group of compounds resembling sterols which are known to stimulate hormonal activity in ovaries, thyroid and pancreas via pituitary gland and hypothalamus. Also, alkaloids, non-steroidal compounds present in Maca-GO, which, although acting much more slowly than soy and clover extracts, are much better tolerated by women remaining on Maca program (5) and positively affect physiological actions along the hypothalamus - pituitary gland – gonadal axis. Maca was successfully used to wean women off hormone replacement therapy (some of them had been on HRT for many years), and for those women who still had some symptoms, a combination protocol involving Maca extract and a minute amount of natural estrogen together with natural progesterone was suggested, which, unlike progestin, is not considered to be carcinogenic (5).

### The effect of Maca-GO on individual menopausal symptoms

Significant increase in E2 level observed in this study after two months of Maca-GO treatment was associated with alleviation of a number of individual menopausal symptoms, most distinctive being reductions in frequency and severity of hot flushes, excessive sweating, interrupted sleep pattern, nervousness depression, headaches and loss of libido/interest in sex (Table 5 and 6). Studies reported by other authors (2, 21) also indicate that Maca can be helpful in reducing discomfort caused by menopausal symptoms. There was a distinctive effect of the length of Maca-GO treatment on the degree of reduction in severity of individual symptoms, with two months of treatment magnifying the positive results already observed after one month of treatment. (Figure 3).

Demonstrated in this study, the positive effect of Maca-GO on lowering E2, and alleviation of the menopausal symptoms not restricted to hot flushes and night sweating only, shows Maca-GO as a natural non-hormonal treatment, superior to HRT in terms of all the additional benefits such as simultaneous reduction in FSH and an increased HDL, not delivered by HRT programs or phyto-estrogenic preparations.

### Comparison of sensitivity of the GMS and KMI tests

Comparing degree of statistical significance in influences of Maca-GO on KMI and GMI in terms of detected reduction in frequency and severity of menopausal symptoms, it appears that KMI is more sensitive in displaying the effect of both Placebo and Maca-GO treatments on the recorded scores of menopausal tests as compared to GMS (Table 7).

### Responses of women to Maca-GO treatment in clinics located in two distant regions

In early- postmenopausal stage, women experience noticeable and unpredictable waves of discomfort with differing intensity of complex physical and psychological manifestations. These are expressed differently by individual patients-subjects and/or groups of women of different lifestyles and/or ethnic backgrounds etc. Results observed in this multi-centre study in women volunteers attending clinics in two geographically distant locations, which differed greatly in both socio-cultural and economic terms, may support such an assumption. Apparent differences were reported (22) in responses of women to HRT due to geographical location (Europe and USA) – the fact, behind a decision made in this study to observe clinical, physiological and vasomotor responses of postmenopausal women to non-hormonal phyto-preparation, as an alternative to HRT, in two geographically-distant locations representing different socio-economic and cultural matrix of participants. There were significant differences in dynamics of E2 responses observed in individual sequence groups to Maca-GO and Placebo treatment. In addition to the effect of Placebo or Maca-GO treatment, responses recorded in individual groups may be associated with participants attending different clinics, located in two geographically-distant regions representing the different cultural and socio-economic population matrixes characterizing Poznan and Glogow.

Although it could not be statistically tested in this study, it may be assumed that different responses to Maca-GO treatment may be confounded by differences in dietary habits amongst populations linked to two regions and groups of women participating in the study (preference for potato-based dishes with fewer leafy vegetables in Poznan and pasta or rice with more leafy vegetables and fruits in Glogow). It has to be emphasized that there were no instructions given to participants regarding use of a specific diet or exercise program nor were records of dietary intake kept by participants in this study.

### The effect of Maca-GO on lipid metabolism indices

Lipid metabolism data, observed in Trial I showed a significant effect of Maca-GO on increasing HDL only without significantly (*P*>0.05) affecting CHOL, TRGL and LDL when relating results to Placebo treatment. There was a distinctive reduction in LDL/HDL ratio from 2.7 to 2.3. In previous study on ovariectomised rats (9), serum HDL also increased together with CHOL and LDL with TRGL noticeably – but not significantly reduced. Reported in the literature (1) positive influence of Maca on overall lipid metabolism has not been confirmed in this study, with one exception of an elevated HDL level.

### Further considerations

In addition to the positive effects of Maca-GO on hormonal balance, HDL and alleviation of menopausal symptoms, results presented in this part of the clinical study on early-postmenopausal women, also demonstrated an existence of Placebo effect, residual Maca-GO effect and a necessity for two month treatment period for Maca-GO to display its full therapeutic functionality. In order to confirm the above findings, in the next, concluding part (III) of this clinical study, using a crossover configuration experimental design, levels of gonadal, pituitary, thyroid and adrenal hormones, lipids and key minerals, were measured in relation to changes in severity of menopausal symptoms contributing to overall degree of discomfort felt by early-postmenopausal women after two months of Maca-GO administration.

## CONCLUSIONS

Two months administration of Maca-GO to early-postmenopausal women volunteers, resulted in significant reduction of Placebo-corrected results for FSH (*P*<0.05) with a simultaneous increase in E2 (*P*<0.001) and HDL (*P*<0.05).

Maca-GO treatment resulted in a significant (*P*<0.01) alleviation of menopausal symptoms as expressed by reduction in frequency and severity of hot flushes and night sweating – in particular, followed by reductions in nervousness, mood swings, interrupted sleep pattern, fatigue, stress, headaches depression, and decreased libido.

Maca-GO treatment significantly reduced menopausal discomfort in early-postmenopausal women as measured by total KMI and GMS values (from 22 to 10 and from 18 to 11 respectively).

Toxicity of Maca-GO assessed in a laboratory model, showed its safety for use as dietary supplement for humans at the level determined as LD50>7.5 g/kg BW (on rats) and LD50>15 g/kg BW (on mice) – much higher than the minimum OECD limit LD50>2 g/kg BW.

For Maca-GO to exhibit its significant hormone-balancing and therapeutic effect, it was essential for women to use it continuously during two consecutive months.

Results indicated an existence of “residual effect” of Maca-GO in most measurements taken, after the treatment was withdrawn and replaced by Placebo for one month.

There were significantly different responses of women in E2 to Maca-GO treatment in clinics located in two distant regions which may be attributed to socio-cultural and economic differences.

## DISCLAIMER

This study was conducted and jointly supported by a long-term joint R&D program of the Research Institute of Medicinal Plants (RIMP) in Poznan, Poland and School of Health Study, Charles Sturt University & Therapeutic Research International, Sydney, Australia on medicinal plants and therapeutic preparations originating in Australasia, Oceania and South America. Thanks are due to Ms. Dorota R. Meissner of Therapeutic Research International for assistance in collecting results from clinics and diagnostic laboratories in Poland including help in compilation and formatting data for computation and data processing as well as Ms. Jan Roberts for assistance in editing of the final draft. A supply of a standardized Maca-GO powder by NatureCorp Pty Ltd, Australia for encapsulation in RIMP, is gratefully acknowledged.
